# Complete genome sequence of Oscillospiraceae bacterium MB24-C1, isolated from sewage sludge

**DOI:** 10.1128/mra.00002-26

**Published:** 2026-03-06

**Authors:** Jingjing Zhao, Wanling Qiu, Fuying Li, Guowen Dong, Yin Li, Chao-Jen Shih, Yen-Chi Wu, Chih-Hung Wu, Shu-Jung Lai, Wangchung Xiao, Wei-Ling Zhang, Nanqiang Li, Sheng-Chung Chen

**Affiliations:** 1 School of Resources and Chemical Engineering, Sanming University 66283, Sanming City, Fujian, People’s Republic of China; 2 Fujian Provincial Key Laboratory of Resources and Environmental Monitoring and Sustainable Management and Utilization, Sanming University 66283, Sanming, Fujian, People’s Republic of China; 3 College of Environment and Safety Engineering, Fuzhou University 12423, Fuzhou, Fujian, People’s Republic of China; 4 State Key Laboratory of Photocatalysis on Energy and Environment, Fuzhou University 12423, Fuzhou, Fujian, People’s Republic of China; 5 Department of Engineering Technology Management, International College, Krirk University 67996, Bangkok, Thailand; 6 Bioresource Collection and Research Center, Food Industry Research and Development Institute 63418, Hsinchu, Taiwan, Republic of China; 7 Graduate Institute of Biomedical Sciences, China Medical University 26488, Taichung City, Taiwan, Republic of China; 8 Research Center for Cancer Biology, China Medical University 26488, Taichung City, Taiwan, Republic of China; Nanchang University, Nanchang, Jiangxi, China

**Keywords:** Oscillospiraceae, sewage sludge, anaerobes

## Abstract

Here, we report the complete genome sequence of Oscillospiraceae bacterium MB24-C1 (= BCRC 81428), isolated from sewage sludge of the Sanming Steel wastewater treatment plant, China. The genome comprises 2,952,321 bp with a GC content of 48.1% and was used for further species delineation and comparative genomic analyses.

## ANNOUNCEMENT

**Fig 1:**
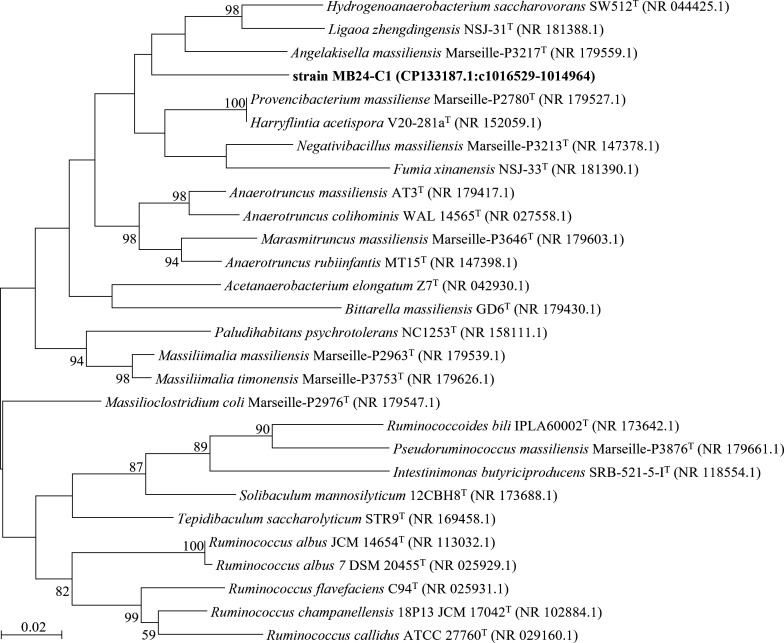
Maximum likelihood tree based on 16S rRNA gene sequences of strain MB24-C1 and related taxa. Bar, 0.02 substitutions per nucleotide position. Bootstrap values were expressed as percentages of 1,000 replications.

Strain MB24-C1 was isolated from dried sewage sludge collected on June 25, 2021, from the Wastewater Treatment Plant (26.2645^o^ N, 117.6245^o^ E) of Sanming Steel Co. Ltd, Fujian, China. The sewage sludge represents a chemically complex and organically enriched anaerobic environment characterized by elevated concentrations of organic matter, nitrogen, phosphorus, petroleum hydrocarbons, and metal elements, which supports diverse anaerobic and fermentative microbial communities. The sample of sludge collected using a 3 mL syringe was inoculated into the anaerobic modified DSM 924 medium (per liter: 1 g MgCl_2_·7H_2_O, 0.5 g KCl, 0.1 g CaCl_2_·2H_2_O, 0.4 g K_2_HPO_4_, 1 g NH_4_Cl, 10 mL trace element solution, 2 g yeast extract, 2 g tryptone, 0.5 ml Na-resazurin solution [0.1%], 4 g NaHCO_3_, 10 ml vitamin solution, 0.25 g L-cysteine-HCl·H_2_O, 0.25 g Na_2_S·9H_2_O, headspace: N_2_:CO_2_=4:1), prepared according to the instructions of the medium and incubated at room temperature (~ 25°C) for 2 weeks. Strain MB24-C1 was purified by serial dilution and the rolling-tube technique (1) using the modified DSM 924 medium at 25°C. The isolate was identified by 16S rRNA gene clone sequencing with 8F (5′-AGAGTTTGATCCTGGCTCAG-3′) and 1492RU (5′-TTTTAATTAAGGTTACCTTGTTACGACTT-3′) primers (2). The purity of bacteria was confirmed by morphological observation, 16S rRNA gene analysis, and genome sequencing. Based on BLASTN analysis (3), the 16S rRNA gene sequence of strain MB24-C1 (from genome, CP133187.1:c1016529-1014964) showed the highest similarity (92.71%) to *Hydrogenoanaerobacterium saccharovorans* SW512^T^ (NR_044425.1) (4). Phylogenetic analysis of 16S rRNA gene sequences performed by MEGA11 (5) for strain MB24-C1 and related taxa indicated that strain MB24-C1 could be affiliated with a novel genus (Fig. 1). The genome of strain MB24-C1 was selected to sequence for the species delineation and comparative genomic analysis.

The strain MB24-C1 has been deposited in the Bioresource Collection and Research Center, Taiwan, as strain BCRC 81428. It was grown in the modified DSM 924 medium and incubated at 30°C. The genomic DNA of strain MB24-C1 was extracted using the NucleoBond HMW DNA kit (Macherey-Nagel, Germany) according to the manufacturer’s instructions. The genome was sequenced at Sangon Biotech (Shanghai) Co., Ltd. using the DNBSEQ-T7 platform (MGI Tech Co., Ltd.) and MinION sequencer (Oxford Nanopore Technology).

For the DNBSEQ-T7 platform, sheared genomic DNA fragments of approximately 300 bp were utilized to prepare a 150 bp paired-end DNA library. The library preparation was performed using HiEff NGS MaxUp II DNA Library Prep Kit for Illumina (Yeasen Biotechnology (Shanghai) Co., Ltd.). The constructed library was sequenced using MGISEQ-2000RS High-throughput Sequencing Set (PE150 format), and 11,365,696 reads were generated (Table 1). Raw sequencing data were processed using Trimmomatic v0.36 (6) to remove ambiguous bases, trim adapter sequences, eliminate low-quality bases (Q < 20) from both ends using a 5 bp sliding window, and discard reads shorter than 35 nt and their paired reads. For the MinION sequencer, a ligation-based library (SQK-LSK109) was prepared from 1 µg of unsheared genomic DNA (>1 kb) and sequenced on an R9.4.1 flow cell. MinION reads were base-called using Guppy v6.5.7 in high-accuracy mode. Raw reads were trimmed with Porechop v0.2.4 (7) and filtered using NanoFilt v2.8.0 (8) to remove low-quality and short reads, resulting in 411,995 reads with an average length of 3,391 bp, an *N*_50_ of 9,397 bp, and a total yield of 1,397,109,541 bp (Table 1). Hybrid reads were *de novo* assembled using Canu v2.2 (9), which identified overlaps, trimmed contig ends, and confirmed circular topology without rotating the genome. The average short-read and long-read coverages were 577× and 473×, respectively. Gene predictions and annotations were performed using the NCBI Prokaryotic Genome Annotation Pipeline (PGAP) v6.5 (10).

**TABLE 1:** Genome characteristics of Oscillospiraceae bacterium strain MB24-C1

Characteristic	Value
Sequencing and assembly features	
No. of MGI-DNBSEQ-T7 total reads	11,365,696
No. of nanopore total reads	411,995
Total no. of MGI-DNBSEQ-T7 bases	1,704,854,400 bp
Total no. of nanopore bases	1,397,109,541 bp
Genome coverage (MGI; Nanopore)	577×; 473×
Genome features	
Chromosome size (GC content)	2,952,321 bp (48.1%)
Total no. of genes	2,832
No. of coding sequences	2,766
No. of pseudogenes	24
No. of rRNAs	9
No. of tRNAs	53
No. of noncoding RNAs	4

The assembly generated a single large contig of 2,952,321 bp with 48.1% GC content, which was circularized by aligning both ends of the contig sequences and deleting overlapped sequences of one end. The genome contains 9 rRNA genes and 53 tRNA genes (Table 1). Default parameters were used except where otherwise noted.

Strain MB24-C1 encodes multiple sporulation proteins (SigG, WMJ83897.1; SpoIIIAC, WMJ84247.1), flagellar components (FliC, WMJ82836.1; FlhA, WMJ82907.1), and a [FeFe] hydrogenase (WMJ83997.1), indicating a motile, spore-forming, and fermentative lifestyle. Key enzymes involved in acetate and butyrate production, including acetate kinase (WMJ83561.1) and butyrate kinase (WMJ84209.1), support its potential application in anaerobic digestion, organic waste degradation, and bioenergy recovery.

## Data Availability

The genome sequence of strain MB24-C1 has been deposited in GenBank under accession number CP133187. The version of the genome described in this paper is the first version. The BioProject and BioSample accession numbers are PRJNA1006091 and SAMN37009672. MinION and DNBSEQ-T7 raw reads were deposited in the Sequence Read Archive (SRA) under accession numbers SRR27217536 and SRR27217537, respectively.
